# Designating river environments as bathing waters: challenges and opportunities at the science-policy interface

**DOI:** 10.1093/sumbio/qvaf015

**Published:** 2025-07-26

**Authors:** David M Oliver

**Affiliations:** Biological and Environmental Sciences, Faculty of Natural Sciences, University of Stirling, Stirling FK9 4LA, United Kingdom

**Keywords:** bathing water regulations, combined sewer overflows, *Escherichia coli*, faecal pollution, public health, river monitoring

## Abstract

Regulatory standards for primary contact recreation are used around the world to limit risks to human health from exposure to poor water quality. Prior to 2020, no stretches of river in the UK were designated as bathing water environments. However, growing recreational use of rivers combined with grassroots action by campaign groups to promote a river clean-up agenda has helped to establish, and increase the number of, designated riverine bathing waters in the UK. Nevertheless, since 2020 most newly designated riverine bathing waters have struggled to meet the microbiological standards associated with the Bathing Water Directive due to anthropogenic inputs originating from farming, sewage, and wastewater discharges. This is, in part, due to UK rivers being managed historically for different ecosystem services, with no statutory monitoring for public health. Calls are growing for river water quality improvements, fuelled by frequent media coverage concerning sewage overflows into freshwater environments and widespread recognition of poor delivery on targets for UK waterbody quality. In response, a five-point plan to improve understanding and future management of faecal indicators at riverine bathing water sites is proposed, offering transferability to other regions of the world where microbiological monitoring of river environments is needed to support recreation.

**Sustainability Statement**

The sustainable management of water resources and effective monitoring of microbial compliance parameters are of fundamental importance for ensuring healthy bathing water environments. The underpinning science and policy that supports the management of designated bathing waters link strongly to SDG6 (clean water and sanitation). To support sustainable management of rivers as designated bathing waters in the UK, there is an urgent need to improve monitoring and modelling capability of microbial compliance parameters. This will help deliver improved health and wellbeing opportunities for recreational users and generate revenue for local economies.

## Introduction

Globally, human population pressure and climate change are impacting on the microbiological quality of surface waters (Seymour and McLellan 2025). Regulatory standards for primary contact recreation (e.g. swimming) are used around the world to limit risks to human health from exposure to poor water quality (Farrell et al. 2021, Adhikary et al. 2022). In Europe, those regulatory standards are set by the European Union (EU) Bathing Water Directive (BWD), which is designed to safeguard public health and protect water environments with designated status from microbial pollution. The microbiological standards used in the BWD reflect epidemiological evidence that informs the World Health Organization (WHO) guidelines for recreational waters, which were established for primary contact recreation involving direct immersion in water (Kay et al. 2004). Recreational exposure to poor bathing water quality, and associated microbial contamination, increases the risk of illnesses that can include gastrointestinal disease, skin and eye infections, and respiratory illness (Tiwari et al. 2021). The implementation of the BWD across EU member states and the UK therefore provides the regulatory basis for managing faecal pollution risks to human health at over 21 000 designated bathing waters, which span marine (coastal) and inland (lacustrine and riverine) sites.

The EU BWD was first introduced in 1976 with bathing water sites designated on the basis that they are popular for swimming or paddling. Regulations have been updated over time to reflect changes in the evidence base used to support the BWD (Oliver et al. 2014) and the UK government transposed the 2006 BWD into national law via the bathing water regulations, becoming ‘retained EU law’ following the UK’s withdrawal from the EU. The UK has over 600 designated bathing waters and the majority of these are coastal. Prior to 2020, no stretches of river in the UK were designated as bathing waters, with only coastal and inland lake sites monitored under the bathing water regulations. In 2020, the first UK riverine bathing water was designated, aligning the UK with many member states of the EU that already monitor sections of river environments within their bathing water assessments. The recent designation of UK rivers as bathing waters has been driven by a shift in wider recreational use of rivers, influenced by growing recognition of the value of local blue spaces since the COVID-19 lockdowns (Whitty et al. 2022). Grassroots action by campaign groups and river trusts to promote a river clean-up agenda has also contributed to increased designation at river locations (Collins et al. 2023). In 2022, a House of Commons Select Committee report on river water quality in the UK recommended that there should be one designated river bathing water per water company area by 2025 (House of Commons Environmental Audit Committee 2022), which would help to provide ‘blue-health’ opportunities to a wider population by increasing access to those living inland. The number of UK riverine bathing waters is therefore increasing, and in 2024 the UK government announced a further 12 rivers in England among a list of new bathing water designations, increasing the total number of UK riverine bathing waters to 15 sites (Defra 2024). A continued increase in designation of riverine bathing waters is expected.

Upon designation, environmental regulators are required to test the microbiological quality of water at bathing sites throughout a defined bathing season. To date, most newly designated riverine bathing waters in the UK have struggled to meet the microbiological standards associated with the BWD due to anthropogenic inputs originating from farming, sewage, and wastewater discharges (Swiminfo 2025). Following the 2024 bathing water season, a disproportionately high number of riverine bathing sites received a ‘poor’ classification, failing to achieve the minimum standard considered acceptable for bathing. This is, in part, due to UK rivers being managed historically for different ecosystem services [e.g. fishing (a secondary contact form of river recreation) and water abstraction] with no statutory monitoring for public health (Whelan et al. 2022). Consequently, UK rivers have had infrequent and disparate sampling campaigns to inform on microbial water quality, as inferred through microbial compliance parameters known as faecal indicator organisms (FIOs), specifically *E. coli* and intestinal enterococci (IE). End-point receptors such as coastal bathing waters, which are monitored for FIOs, have instead served to indicate catchments with faecal pollution pressures; however, coastal waters integrate and dilute pollution signals from the wider catchment contributing area, allowing individual stretches of (potentially problematic) rivers to go unnoticed in terms of regulatory monitoring of faecal pollution issues (Winter et al. 2011). Good quality national-scale data to inform understanding and underpin targeted sustainable management of riverine bathing water quality is therefore lacking (Oliver et al. 2016, Krupska et al. 2024).

In the 1990s, the drive to improve coastal water quality led to substantial improvements in bathing water quality surrounding the UK coastline (Kay et al. 2004). Three decades later and calls are growing for river water quality improvements, fuelled by frequent media coverage concerning sewage overflows into freshwater environments and widespread recognition of poor delivery on targets for UK waterbody quality (OEP 2024). While relatively few of the recently designated river bathing waters are meeting the minimum (sufficient) BWD standards for bathing, their new bathing water status and accompanying regulatory attention should act as a catalyst to help foster the coordinated action needed to improve microbial water quality of these rivers. Elsewhere, calls are growing for removal of bans implemented in the 20th century on *urban* river swimming in major European cities (Kistemann et al. 2016), signalling wider international interest in managing rivers for bathing. The designation of rivers as bathing water environments in the UK therefore provides opportunities for focusing improvements; however, researchers, regulators, and policy practitioners concerned with managing bathing water quality in these dynamic environments face challenges given limited availability of microbial water quality assessments of river environments to date.

## Bridging the knowledge gap in riverine bathing water and faecal indicator research

### Threats to the microbiological quality of rivers

Human wastewater discharges and agricultural runoff are the two sources responsible for the majority of faecal pollution load in rivers (Cheung et al. 2025). However, unlike other water quality parameters such as nutrients, e.g. phosphorus and nitrogen, spatially distributed data on FIO concentration and load within catchment drainage networks are scarce (Whelan et al. 2022). Further, while catchment land use is recognized to influence FIO load in rivers, in-stream evidence to inform on the relative contribution of FIOs from human versus agricultural sources is limited. In large mixed land-use catchments where FIO contributions can potentially arise from a complex mix of diffuse agricultural sources and point source discharges from wastewater treatments plants (WWTPs), combined sewer overflows, and leaking septic tanks, effective risk management of designated river bathing waters will therefore be challenging (Tipper et al. 2024, Suslovaite et al. 2024). The use of microbial source tracking (MST) data offers scope to gauge relative contributions from different catchment sources of faecal loading; however, MST data only provide a snapshot of the relative contribution of human versus livestock-derived FIO sources for a particular point in time, and signature of river water quality can be dynamic over relatively short timescales depending on catchment management, antecedent weather conditions, and river dynamics (Karunakaran et al. 2024, Murphy et al. 2025).

### Hidden risks to river water quality from combined sewer overflows?

Sewage overflows to UK surface waters are at unacceptably high levels, with frequent media reports of excessive spills of raw sewage raising public awareness of the risks posed to water quality and downstream ecological and public health (House of Commons Environmental Audit Committee 2022, Ford et al. 2025). Extreme rainfall can challenge the efficiency and reliability of the sewerage infrastructure designed to manage wastewater transfers and subsequent treatment. Combined sewer overflows (CSOs) are part of the sewerage infrastructure associated with WWTPs; they are designed to spill untreated human sewage in ‘exceptional’ circumstances, i.e. very wet weather, when WWTPs become overwhelmed with stormwater. Reductions in wastewater treatment efficiency during extreme wet weather events can have localized and downstream consequences in terms of risk to the environment, ecosystem service provision, and human health (Perry et al. 2024). At the end of 2024 there were 14 254 active storm overflows operating in England, with spill data available for 14 182 of these discharge points. In total, 450 398 spills were recorded spanning a combined duration of 3.6 M hours through 2024. This equates to, on average, 31.8 spills per recorded overflow with an average of 8 hours per spill, although this data lacks information on volumes of sewage discharged (Environment Agency 2025). These statistics have attracted strong criticism from river conservation groups, water quality experts, and public health professionals who are increasingly concerned that CSOs are being used to regularly dispose of untreated sewage into receiving waters even during times of little to no rainfall, so-called ‘dry spilling’. This has been coupled with a series of record fines for some UK water companies for major sewage leaks and other pollution incidents (Hammond et al. 2021).

While the immediate consequences of sewage spills to water quality are well recognized, there are secondary concerns associated with the potential accumulation of solid fractions of untreated human sewage in riverbed sediments (Oliver et al. 2024). Depending on factors such as river flow, microbial-particle associations, and sedimentation rates, a proportion of faecally derived microbial pollutants will become incorporated along with solids in the riverbed (Donovan et al. 2008, Hachad et al. 2022). Consequently, there is potential for residual stores of FIOs to build up over time in riverbed sediments near to where CSOs discharge, representing hidden and transient storage zones (Devane et al. 2014). There is a growing body of research that has documented the delayed impacts that residual or ‘legacy’ phosphorus can have on water quality and the associated implications for catchment management (Sharpley et al. 2013, Shober et al. 2025); however, the importance of legacy risks associated with transient storage zones of FIOs in sewage-contaminated riverbed sediments has received much less attention.

There is also potential for FIOs to associate with microplastics discharged into rivers via wastewater effluent (Metcalf et al. 2023). Furthermore, sewage debris from CSOs often contains plastic, such as wet wipes, sanitary products, and cotton bud sticks, which have been shown to harbour FIOs (Metcalf et al. 2022). These plastics may be retained in the riverbed following sedimentation, become trapped in bankside vegetation, or wash up on riverbanks and the terrain adjacent to the riverbed (Nyberg et al. 2023, 2024, Gallitelli et al. 2025). Plastic debris and litter trapped in and around the river channel therefore represents another potential transient or residual store of FIOs capable of delayed impairment of downstream river bathing water environments. However, their role in contributing to both immediate and long-term impacts on bathing water environments remains poorly understood (Oliver et al. 2024).

### Data and modelling needs

Limited understanding and data, coinciding with a drive to increase the number of UK rivers designated with bathing water status, highlights a pressing knowledge gap that needs addressing to help manage UK rivers for public health. Growing interest in river recreation is leading environmental regulators and decision-makers to rely on modelling capability in the absence of national FIO river monitoring data to identify spatial and temporal drivers of FIO risk to river environments and to help understand potential microbial pollution dynamics at locations designated for bathing (e.g. Hankin et al. 2019). Models can inform on likely in-stream FIO concentrations and loads, helping to understand FIO source apportionment and faecal pollution risk at end-point receptors (and likely compliance with the BWD standards). Modelling also underpins information displayed on electronic signage during the bathing season at a subset of coastal and lacustrine bathing water locations across the UK. Such information, updated daily, includes advice against bathing when water quality is predicted to fall below the sufficient microbiological threshold (Krupska et al. 2024). While the extension of electronic signage to river bathing waters would be welcome, underpinning predictive capability would be challenged due to the sub-diurnal patterns of river FIO concentrations and their susceptibility to short-term pollution fluctuations. Riverine bathing water signage would at the very least need to be more responsive through the course of a bathing day. Tools and approaches to predict *E. coli* concentrations and loads in rivers have been developed; spatial mapping and stream-network tools can identify in-stream risk of *E. coli* pollution (e.g. Dymond et al. 2016, Neill et al. 2018) and process-based models have evolved over time to reflect up-to-date parameterization (e.g. HYPE; Hankin et al. 2019, Srinivasan et al. 2021). However, modelling effort alone will not deliver a ‘silver bullet’ to compensate for our limited understanding of riverine FIO dynamics; models rely on observed data to underpin their development, with the quality of predictions reliant on the quality of empirical data used to inform each model (Oliver et al. 2016).

Critically, modelling effort to date has focused on *E. coli*, rather than IE, as the FIO of choice. This is likely due to parameterization data for microbial pollution models (e.g. FIO catchment inputs; FIO fate and transfer behaviour in different environmental matrices) being more readily available for *E. coli*. In contrast, relatively limited field-relevant data are available to support IE model development and associated parameterization, calibration, and testing (Oliver et al. 2012, 2010). This exposes shortcomings in existing modelling frameworks used in the UK for predicting riverine bathing water quality; environmental regulators in the UK cannot rely on models of a single microbial compliance parameter in assessing how different management scenarios might impact on bathing water compliance at potential future river sites. This is because both FIOs are used as microbial compliance parameters within the bathing water regulations (Kay et al. 2004, Table 1).

**Table 1.** Microbial compliance parameters and their threshold values for determining bathing water classifications at coastal and inland (lacustrine and riverine) sites, as outlined in the Bathing Water regulations.

Bathing water location	Microbial compliance parameter	Bathing water classification	Threshold (CFU/100 mL)/(percentile)
Coastal (marine water)	*E. coli*	Excellent	≤250 (95th percentile)
		Good	≤500 (95th percentile)
		Sufficient	≤500 (90th percentile)
		Poor	Sufficient standard not met
	Intestinal enterococci	Excellent	≤100 (95th percentile)
		Good	≤200 (95th percentile)
		Sufficient	≤185 (95th percentile)
		Poor	Sufficient standard not met
Inland (fresh water	*E. coli*	Excellent	≤500 (95th percentile)
		Good	≤1000 (95th percentile)
		Sufficient	≤900 (90th percentile)
		Poor	Sufficient standard not met
	Intestinal enterococci	Excellent	≤200 (95th percentile)
		Good	≤400 (95th percentile)
		Sufficient	≤330 (95th percentile)
		Poor	Sufficient standard not met

CFU: colony forming unit.

The assessment is based on a percentile evaluation of the log_10_ normal probability density function of microbiological data.

### National-scale surveillance monitoring of FIOs in UK rivers: a ‘thought experiment’

There is no national-scale surveillance monitoring of FIOs in UK rivers. If unconstrained by resources, a baseline level of national-scale river monitoring for FIOs, initiated as soon as possible and irrespective of river bathing water status, would in time bring about a step-change in our ability to understand opportunities and threats for future bathing water designation at river sites around the UK. Surveillance monitoring of this nature, established over multiple years to recognize year-on-year hydrological variability, would deliver many potential benefits (Werner 2025). For example, it would enable a ‘health-check’ of the UK river network regarding faecal pollution and allow patterns of FIO responses to be identified according to geographical location, land use, climatic variables, and other environmental factors, in turn aiding management and modelling of bathing waters (both on rivers but also downstream at the coastal environment too). Implementing national-scale FIO monitoring of the UK river network would, however, represent a significant undertaking and highlight nontrivial issues around: (1) frequency of sampling to support a baseline level of understanding representative of different river flow conditions for meaningful interpretation; and (2) where to sample (both along the river network, but also within the specific channel at any identified sampling locations) in order to deliver representative insight into microbial water quality of river environments (see McDowell et al. 2024).

While the idea of national-scale FIO monitoring in rivers is an interesting hypothetical scenario, full-scale implementation by UK environmental regulators seems unlikely. However, questions relating to spatial and temporal characterization of the microbiological quality of rivers remain pertinent to discussions on how to best approach effective microbiological monitoring and management of current and future designated riverine bathing waters. Without historical river surveillance monitoring for FIOs, and operating within the constraints of regulatory funding budgets, there needs to be greater focus on enhancing existing knowledge and modelling capability to cost-effectively support our understanding and sustainable improvement of bathing water environments established on rivers.

## Charting new waters: recommendations and future opportunities at the science-policy interface

Increasing pollution pressures under environmental change and the growing trend in designating bathing waters on UK rivers present a paradoxical perfect storm. Effective management of faecal inputs into river systems would help to mitigate rising pollution pressures from both human and agricultural sources; however, this relies on greater compliance among both water and sewerage companies and the farming community with respect to their operational discharges to receiving waters. UK rivers are increasingly being used for recreation irrespective of designated bathing water status, but opting out of river designation would be counterproductive, reducing pressure on upstream industries to safeguard microbial water quality for downstream users. Given the above, a more pragmatic approach to support sustainable management of rivers as designated bathing waters would be to consolidate, and improve, our understanding of riverine FIO fate and transfer dynamics and in turn mitigate the ‘perfect storm’ arising from the convergence of rising faecal pollution pressures and increasing potential for human exposure via polluted leisure (Evers and Phoenix 2022). This raises important questions: what can we do to improve our understanding of river microbiological quality and better inform inland bathing water management, regulation, and policy? What progress can we make in our monitoring and modelling of river environments to capitalize on opportunities and mitigate challenges linked to future river bathing water designations? In response to these questions, a five-point plan is outlined to improve understanding and future management of FIOs in riverine bathing waters (Table 2). Details of the underpinning need for each recommendation are provided in the following sections.

**Table 2.** Five-point plan to improve understanding and future management of FIOs in riverine bathing waters.

#	Recommendation	Key requirements
1	Consolidate existing piecemeal FIO data	Review, cross-compare, and integrate data, including regulatory, academic, and citizen-science sources.
2	Strengthen FIO modelling capability for river environments	Capitalize on **#1;** value different model types, including risk-based approaches; identify key model parameterization needs; consider expert judgements in absence of data.
3	Recognize legacy impacts from residual and transient FIO stores	Characterize riverbed sediment ‘source-sink’ relationships with FIOs; evaluate risks to bathing water quality from the freshwater plastisphere.
4	Account for spatial and temporal variability of FIOs in rivers	Characterize spatial and temporal variability of FIOs in different river types and conditions; design monitoring to better reflect identified variability.
5	Learn from international best practice	Identify global partners with track record in monitoring of microbial compliance parameters in rivers to meet regulatory requirements.

### Consolidate existing piecemeal FIO data

Relative to the UK, river bathing is more popular in countries such as France (>400 river bathing waters) and Spain (>150 river bathing waters) (EEA 2024). However, the wild swimming movement in the UK, along with a growing public distrust of water and sewerage companies and their excessive use of CSO discharges, has helped raise the importance of river water quality (Armitage 2022). Low numbers of river designation highlight the historical lack of interest in the UK to monitor rivers for public health. This is a significant oversight that is now converting into a challenge in the face of growing interest in river bathing; the UK has seemingly sleptwalked into a situation of having limited riverine FIO concentration and load data to support future management of bathing waters designated on river sites. Across the UK there exist piecemeal data on FIO concentrations in rivers and environmental regulators and those with a responsibility for managing bathing water environments need to act quickly to make the most of what data are currently available. Integrating existing datasets could help to identify future sites more suitable for river bathing and underpin better management efforts at designated river locations already struggling to meet required standards. Existing data have been derived via disparate sampling programmes collected via regulators (e.g. catchment blitz monitoring in priority catchments of concern following pollution incidents and repeated failing of standards at coastal bathing waters) (Akoumianaki et al. 2020), researchers (e.g. specific projects funded to investigate FIO fate and transfer in catchments) (Oliver et al. 2009, Kay et al. 2018), and the public (e.g. citizen science data) (Collins et al. 2023). Collating and consolidating these datasets would be a valuable exercise but would be accompanied with challenges. This would include, for example: (i) navigating different levels of data ownership and access, and the potential for data sharing agreements in some cases; (ii) understanding the comparability of data given the potential for different methods of microbiological analysis used to determine FIO concentrations; (iii) quality control checks to attribute confidence to data derived from different sources; (iv) ensuring access to contextual information such as river flow, rainfall data, and orders of stream and river reaches monitored; and (v) acknowledging limitations with what will mostly be historical rather than contemporary datasets.

### Strengthen FIO modelling capability for river environments

Current understanding of how to best manage designated river bathing waters is constrained by piecemeal empirical data, as described above. This results in greater reliance on modelling capability among environmental regulators and decision-makers. However, managing and modelling the zone of impact (e.g. bathing water) is not the only strategy that environmental regulators can deploy to protect water quality standards. Identifying and managing spatially distributed pollutant hotspots within a catchment can also help to alleviate their cumulative impact at a downstream bathing water, whether riverine or coastal (Dymond et al. 2016). For example, the identification of particular ‘at risk’ tributaries within a catchment can focus awareness-raising campaigns and target mitigation and management advice in areas likely to generate the largest water quality improvements relative to the resource invested. Resources available to environmental regulators are limited; hence, models and tools capable of predicting the distribution of pollution risk across large landscape scales offer a means of prioritizing effort and targeting scarce resource more efficiently and effectively (Neill et al. 2018). In response, advances have been made in the UK to develop risk-based tools for assessing in-stream risk of FIO pollution (Porter et al. 2017, Oliver et al. 2018). With questions increasingly focusing on when and where to prioritize spatial targeting of mitigation and management within catchments, there is a need for models that take a risk-based approach (e.g. Reaney et al. 2011, Glendell et al. 2022).

Unfortunately, as discussed earlier, existing models of FIO fate and transfer in catchment systems are not well equipped to deal with IE. An increased focus on IE and associated fate and transfer behaviour, observed across multiple scales and different environmental matrices and land-use settings, would provide much needed data to help parameterize FIO models capable of assessing both *E. coli* and IE risks within river environments, as required for bathing water classifications. Appreciating that new data are associated with time and cost constraints an alternative, albeit less attractive, approach would be to explore the feasibility of converting existing *E. coli* models to IE equivalents. For example, systematic review and meta-analysis could inform on whether, and by how much, existing *E. coli* model coefficients and parameters would need to vary if predicting IE rather than *E. coli* behaviour in the environment. Standardized expert elicitation protocols (e.g. Mzyece et al. 2024) could also be used to derive expert judgements, and associated probability density functions, on converting model parameters from *E. coli* to IE equivalents in the absence of empirical data (e.g. likely differences in FIO behaviour as informed by half-life/T_90_ values in wastewater, faeces, soil, and water; on-farm die-off; daily shedding rates from different livestock types, etc.). Systematic review, meta-analysis, and expert judgements (acknowledging uncertainty) would offer complementary information to then support ‘first approximation’ *E. coli* to IE parameter conversions to embed into existing modelling capability prior to new data being collected.

### Recognize legacy impacts from residual and transient FIO stores

Rivers are vulnerable to episodes of short-term pollution following storm events or drought conditions (Muirhead and Meenken 2018, Seis et al. 2024). Dynamic flow conditions in river environments can also encourage sediment resuspension, which may in turn deliver FIOs back into the water column as freely suspended cells or associated with resuspended organic and inorganic materials from the riverbed (Muirhead et al. 2004, Cho et al. 2016). Currently, we know relatively little about how such potential residual FIO stores in sewage-contaminated riverbeds vary in terms of their spatial and temporal characteristics or how the survival and resuspension of FIOs from these hotspots can contribute to subsequent downstream risk, including the potential for impacting on bathing water quality, whether that be at a designated riverine, lacustrine, or coastal bathing water sites. These transient FIO stores may be particularly important downstream of CSOs. With increasing attention focusing on the relatively acute water quality impacts of sewage spills, it would be prudent to also recognize and quantify the longer-term risks that CSO discharges can contribute via the stockpiling of FIOs in riverbed sinks downstream of CSOs. Through erosion and resuspension processes associated with shear stress theory, hyporheic exchange, and bioturbation, these sinks or residual stores may then impact on downstream river bathing sites; however, the legacy impacts of FIO resuspension are likely to be spatially and temporally variable due to riverbed heterogeneity and changes in flow dynamics, making meaningful regulatory monitoring difficult via one-spot samples typically used for bathing water assessments.

Effective management of riverine bathing waters therefore requires a better understanding of the circumstances under which riverbed sediments act as a source or sink of FIOs and whether this varies seasonally or spatially as a function of proximity to WWTPs and CSOs, but also due to other factors such as underlying geology and surrounding land use. Resuspension opportunities for FIOs will not only be driven by hydrological factors; slow-flowing bathing water zones on rivers will attract bathers and paddlers, whose activity will disturb the bed sediment (Pattis et al. 2024). Resuspension of sediments currently receives little attention in terms of the potential impacts to bathing water quality; this action is likely to be much more visible, and of consequence, in river environments relative to coastal environments. A more detailed account of FIO fate and transfer processes at the water-sediment interface would inform on resuspension risks of FIOs associated with different riverbed compartments such as epilithic biofilm, fine sediment, or the hyporheic zone (Devane et al. 2014, Sauvage et al. 2018) and enable a much-needed characterization of the sediment ‘source-sink’ relationship with FIOs.

Legacy impacts of FIOs on river bathing water quality may be associated with other sources, such as sewage-related plastic debris. The colonization of plastic surfaces by microbial biofilm leads to the formation of a ‘plastisphere’ community (Zettler et al. 2013). Plastisphere research to date has largely focused on community composition and how microbial (including FIO) survival and transfer can be sustained and enhanced through biofilm formation on plastic. Future research to understand how FIOs may escape the plastisphere through various detachment processes would be useful. For example, are detachment mechanisms of erosion and sloughing important in facilitating the release of FIOs from plastic debris and how significant might this be with respect to impacting on downstream river bathing quality? (Micro)plastics colonized by FIOs will also accumulate in riverbed sediments near to where CSOs discharge but data on microplastic deposition and burial rates in riverbeds is limited, in turn hindering our understanding of the locations of deposition and the timescales over which burial occurs for microplastics and, by association, their co-pollutants (Arnon 2025). Effective risk management of riverine bathing waters therefore needs to recognize not just the immediate risks from contemporary CSO discharges or agricultural runoff, but also legacy contributions from a range of sources in and around the river corridor. Understanding the importance of the latter requires investigation across scales, ranging from new mechanistic understanding of FIO detachment and resuspension processes under laboratory conditions through to empirical in-stream observation of how sewage-contaminated riverbed hotspots operate as an FIO sink versus source over a variety of river typologies and hydrological conditions.

### Account for spatial and temporal variability of FIOs in rivers

Bathing water monitoring currently requires only one sampling location, yet it is widely acknowledged that FIO concentrations will vary spatially in a waterbody (Jozić et al. 2024), including on different banksides of river corridors (Quilliam et al. 2011). For riverine bathing waters, the current approach undertaken by the Environment Agency in England is to use sampling points on rivers downstream of the designated bathing water zone, which it believes is representative of water that has passed through the entire bathing water zone, accounting for all potential pollution inputs. The validity of this claim is questionable; in some cases, the bathing area identified may span distances exceeding 1 km and therefore potentially accommodate multiple sources of pollution and opportunities for sedimentation and resuspension prior to sample acquisition. Can one downstream sample location be fit-for-purpose in providing microbial water quality information representative of the entire designated bathing zone and how does this reflect short-term temporal fluctuations? Using multiple sampling points to classify bathing water quality was proposed as a wider (future phase) reform in a recent UK government consultation (November–December 2024) on proposals for reforming the Bathing Water Regulations 2013 in England and Wales; in reply, 91% of respondents signalled agreement with the need for this reform (Defra 2025).

Within-day variability in microbial concentrations at an urbanized UK coastal bathing water has also served to highlight implications of using a single compliance sample to characterize bathing water quality over time for regulatory monitoring (Wyer et al. 2018). Similarly, in rivers, short-term fluctuations in water quality are common and riverine environments are vulnerable to short-term contamination events (Wyer et al. 2010). Rivers can also carry signatures that reflect diurnal cycles; for example, diurnal signals in untreated wastewater discharge can remain relatively intact downstream of WWTPs in some river environments (Facchi et al. 2007), thus demonstrating that infrequent sampling, in addition to using only one sampling location, further risks insufficient characterization of river bathing water quality. For some pollutants, alternative approaches include composite sampling over longer timescales (e.g. 24 hours) using automatic samplers; however, regulatory protocol for FIOs requires sterile sampling, limiting the use of automated methods that cannot guarantee aseptic sample acquisition (Kay et al. 2008).

Adopting flow proportional sampling may be advantageous for assessing microbiological quality of river bathing waters rather than using pre-scheduled (time-interval) compliance sampling regimes as currently done for UK bathing water assessments. A series of river sampling trials across different river typologies and under varying flow conditions would deliver evidence to support (or reject) such an approach by, for example, quantifying the difference in FIO concentrations determined via a single spot sample collected once every two weeks versus determining a mean concentration of FIOs as informed by daily or even sub-daily sampling over the same two-week period (and at multiple locations within a designated river bathing zone). Sampling campaign studies of this nature have revealed that infrequent sampling can lead to considerable underestimates for other pollutants (Bieroza et al. 2014, Perks et al. 2017) and are likely to return similar concerns for FIOs. The current approach to bathing water sampling does not appear to be evidence-based given the recognition that both time and location, in addition to number of samples, can influence the reliable assessment of bathing water quality (Jozić et al. 2024).

Deploying *in-situ* sensors to inform on *E. coli* concentrations in real time would deliver very useful information if the values returned were reliable and comparable to standard methods; however, while some sensors exist their performance is variable and thus usefulness under real-world scenarios remains dubious (Suslovaite et al. 2024). In the absence of reliable sensor technology for FIO quantification, effort should be invested in exploring how to design optimal FIO monitoring for informing on riverine bathing water quality. This includes critical assessments as described above with respect to the identification of key sampling locations and associated frequencies of sampling, with recognition that hydrodynamics and FIO fate and transport in catchments will play a role. Furthermore, monitoring should serve multiple purposes, including for compliance (spot samples) and classification (longer-term), in addition to specific investigations of contamination sources. Methodologies have been proposed to aid in such sampling design for river water quality (Jiang et al. 2020), although others argue that responsible management of bathing water quality requires a site-specific approach (Jozić et al. 2024, Murphy et al. 2025).

### Learn from international best practice

Finally, the UK is not pioneering the designation of rivers as bathing water environments; it is lagging many other countries that have invested considerable resources into protecting and enhancing river environments for immersive river recreation and bathing water management. Consequently, there are important lessons to learn from other areas of the world, both in terms of good practice but also from wider challenges faced, e.g. concerns raised about health risks to athletes participating in open-water swimming events and the triathlon in the River Seine during the 2024 Paris Olympics (which reopened for public swimming in 2025). The substantial effort undertaken by many EU member states to monitor and manage river bathing waters represents an obvious network to consult given that the BWD operates as a common policy driver both in the EU and currently within the UK. Elsewhere, environmental regulators in New Zealand and the USA both have a long track record of monitoring microbial water quality in rivers during the swimming season. Those with a responsibility for designating and managing UK rivers as bathing water environments therefore have a wealth of experience to draw on via global partners to help deliver sustainable management of these sites. Learning from international best practice in monitoring and management of microbiological quality of rivers for supporting sustainable recreational opportunities is becoming more and more urgent as numbers of designated bathing waters on UK rivers continue to rise.

## Data Availability

All relevant data are contained within this article.
